# Association between triglyceride glucose body mass index and cardiovascular disease in adults: evidence from NHANES 2011- 2020

**DOI:** 10.3389/fendo.2024.1362667

**Published:** 2024-07-16

**Authors:** Run Wang, Xiaobing Cheng, Weijun Tao

**Affiliations:** Department of Cardiology, The Third People's Hospital of Hefei (The Third Clinical College of Anhui Medical University), Hefei, Anhui, China

**Keywords:** cardiovascular disease, TyG-BMI, insulin resistance, NHANES, cross-sectional studies

## Abstract

**Background:**

The association between insulin resistance and cardiovascular diseases (CVD) is of significant interest. However, there is limited published research on the relationship between CVD and the triglyceride glucose-body mass index (TyG-BMI). This study aims to examine the association between TyG-BMI and CVD in US adults.

**Method:**

We analyzed data from 11016 adults collected through the 2011-2020 NHANES. Employing weighted generalized linear models, subgroup analysis, sensitivity analysis, and receiver operating characteristic curves, we examined the association between the TyG-BMI index and CVD. Nonlinear associations were investigated using restricted cubic splines.

**Results:**

Higher TyG-BMI values were significantly associated with an increased prevalence of CVD (P<0.001). Weighted generalized linear models consistently demonstrated a positive association across all models. Specifically, individuals in the highest tertile of TyG-BMI had a 38% higher CVD prevalence than those in the lowest quartile (OR=1.380; 95% CI=1.080, 1.763). Unweighted logistic regression models further confirmed these findings. Sex, race, education, family income to poverty ratio, smoking, hypertension, and diabetes did not modify this positive association (P for interaction >0.05). Incorporating the TyG-BMI index into traditional risk factor models marginally improved the prediction of CVD prevalence (P for comparison <0.05).

**Conclusions:**

The TyG-BMI index, an indicator of insulin resistance, is significantly positive associated with a higher prevalence of CVD. These findings underscore the importance of managing insulin resistance to prevent CVD and highlight the need for further research into the underlying mechanisms of this association.

## 1 Introduction

Globally, cardiovascular disease (CVD) stands as a primary contributor to adult morbidity and mortality (1). In 2020, approximately 928,741 Americans succumbed to CVD, marking the highest annual death toll in recent years (2). In the US, the financial cost of CVD is enormous, totaling hundreds of billions of dollars annually (2).

Insulin resistance is a pathological condition characterized by reduced sensitivity and responsiveness to insulin’s actions, primarily affecting the metabolism of sugar, fat, and energy (IR) (3–5). The triglyceride glucose body mass index (TyG-BMI) and the hyperinsulinemic-euglycemic clamp technique have comparable specificity and sensitivity, according to recent research (6, 7), providing a simpler and more cost-effective means of assessing IR (8). The TyG-BMI index and diabetes and hypertension have a high relationship in several studies (9–11), especially noteworthy, a substantial relationship with the risk of heart failure in those with diabetes or pre-diabetes (12). Moreover, cohort studies including patients receiving peritoneal dialysis have demonstrated a separate association between elevated rates of CVD morbidity and all-cause death and the TyG-BMI index (13). Despite these results, the connection between CVD in general individuals and the TyG-BMI index is unknown.

Therefore, our study aimed to investigate the association between TyG-BMI and CVD in US adults using data from the 2011 to 2020 NHANES database. The potential of the TyG-BMI index as a novel biomarker for predicting cardiovascular disease in the general population warrants in-depth assessment. This could provide more precise information for the early prevention of cardiovascular disease.

## 2 Method

### 2.1 Study population

An important program of the National Center for Health Statistics (NCHS) is the National Health and Nutrition Examination Survey (NHANES), used to assess the nutritional and general health of adult and pediatric populations in the US.

Due to the complex probability stratified sampling design of the NHANES database, we adhered to the NHANES guidelines for complex sampling weight calculations, to ensure robust and representative statistical analysis. NHANES survey data, detailed experimental methods, and consent forms are available at www.cdc.gov/nchs/nhanes/

In the study, we analyzed data from 45,462 participants from the NHANES database covering 2011 to 2020. We excluded 19,182 participants younger than 20 years old. We excluded 58 participants with missing education levels and smoking status. Furthermore, the analysis excluded 15211 participants due to incomplete questionnaire data and incomplete TyG-BMI data, which was essential for diagnosing cardiovascular diseases and calculating TyG-BMI. Eventually, we employed 11,016 participants in the analysis.

**Figure 1:**
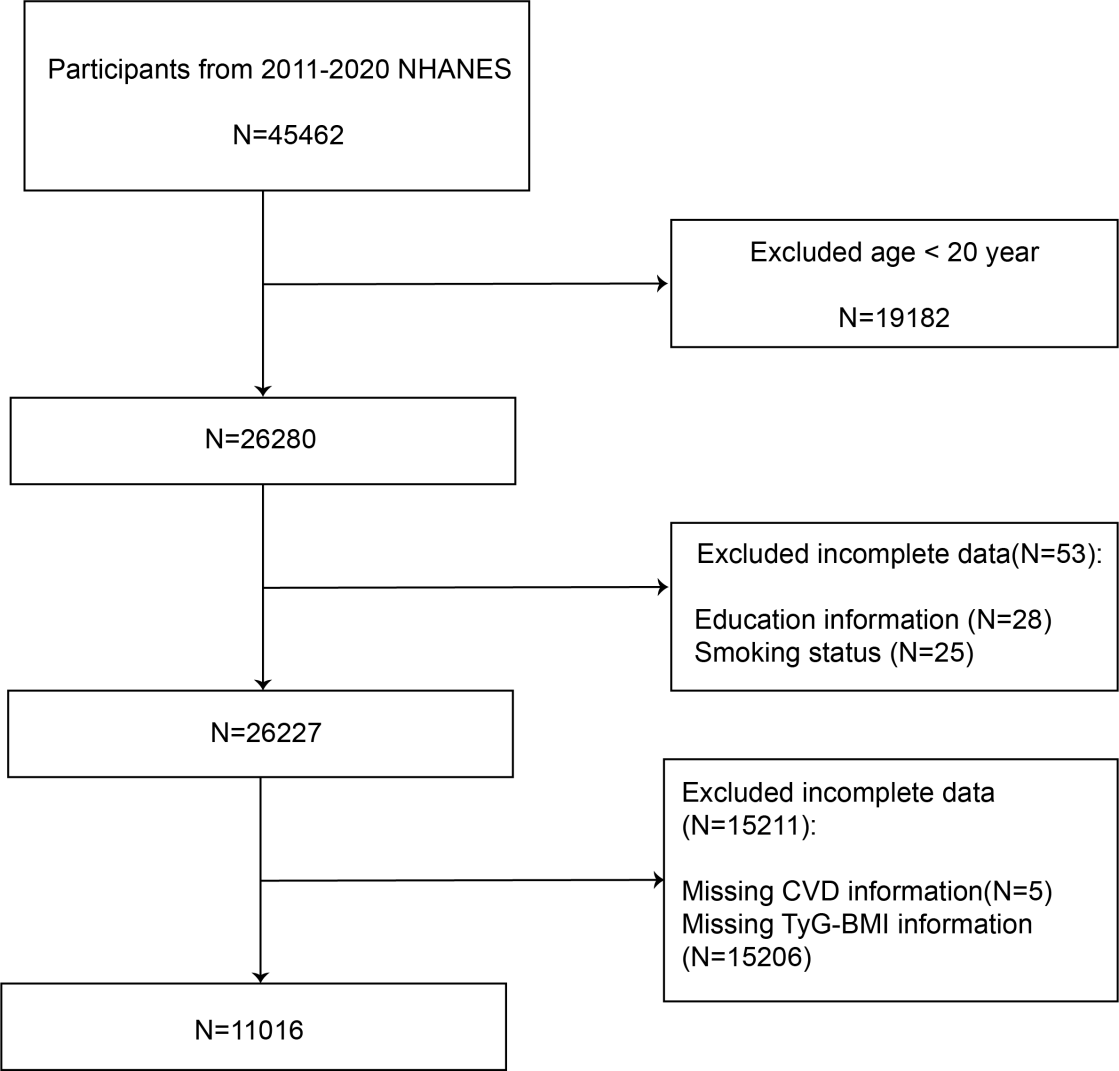
The process flow diagram for the systematic selection method.

**Figure 1** shows a flow diagram for the research individuals and details the methodical process of participant selection.

### 2.2 Definition of cardiovascular diseases

The definition of CVD in this study was derived from survey questionnaire replies. Any of the following conditions reported by a participant were used to classify them as CVD: angina, myocardial infarction (MI), stroke, congestive heart failure (CHF), or coronary heart disease (CHD). Any response that was positive to these questions was thought to be indicative of CVD.

### 2.3 Definition of triglyceride glucose-body mass index


TyG index=Ln [TG(mg/dL)×FBG (mg/dL)/2]

$$\text{BMI}=\text{weight}\;(\text{kg})/\text{height}{\;(\text{m})}^{2}$$

TyG−BMI=TyG index×BMI

The purpose of TyG-BMI in our study was to be an exposure variable (14, 15).

### 2.4 Covariates

A wide range of factors were investigated in this study, such as age, gender, educational achievement, racial background patterns of alcohol and cigarette consumption, and estimated glomerular filtration rate (eGFR). Clinical conditions including hypertension, diabetes mellitus, and hyperlipidemia were also taken into account. Based on educational background, three groups were established: less than high school, high school or its equivalent, and college education or above. Three groups were established based on the family income-to-poverty ratio (PIR): low (PIR<1.3), middle (≥1.3 and <3.5), and high-income (PIR≥3.5) (16). eGFR was computed using the creatinine equation for CKD-EPI (17). Alcohol consumption was defined as excessive at four or more drinks per day for men and three or more for women, moderate at two drinks per day for women and three for men, with any additional intake considered minimal (18). Smoking status was determined by a history of consuming over a hundred cigarettes in a year (19). Hyperlipidemia was defined using specific thresholds: total cholesterol ≥ 200 mg/dL, triglycerides ≥ 150 mg/dL, high-density lipoprotein ≤ 40 mg/dL in men and ≤ 50 mg/dL in women, or low-density lipoprotein ≥ 130 mg/dL (20, 21). Hypertension is defined as the average of the previous three blood pressures with a systolic blood pressure ≥ 140 mmHg, and or diastolic blood pressure ≥ 90 mmHg or a use of antihypertensive drugs or a history of documented hypertension. A fasting plasma glucose level of 126 mg/dL or above, glycated hemoglobin (HbA1c) readings of 6.5% or above, or the use of insulin or hypoglycemic drugs were the criteria used to diagnose diabetes. For the study, every measurable clinical parameter and laboratory result was painstakingly gathered.

### 2.5 Statistical analysis

To ensure national representation, particular sample weights (WTMEC2YR and WTMECPRP), strata (SDMVSTRA), and clusters (SDMVPSU) were applied. We multiply interpolated a portion of the missing data. Continuous variables were reported as means with 95% confidence intervals (CIs), while categorical variables were described as proportions with 95% CIs. Participants were divided into tertiles based on their TyG-BMI for the baseline characteristics analysis. The association between CVD and TyG-BMI was examined using weighted generalized linear models. The first model (Model 1) included no adjustments. The second model (Model 2) adjusted for demographic factors including sex, age, and race. The third model (Model 3) further adjusted for socioeconomic and lifestyle factors such as education, PIR, smoking status, alcohol use, hypertension, hyperlipidemia, and diabetes. Nonlinear associations were explored using restricted cubic spline curves. Weighted subgroup analyses were conducted to evaluate the robustness of the association between TyG-BMI and CVD. Validation of our findings was performed using unweighted logistic regression analysis. To determine if the TyG-BMI index increases the prediction ability of models that include conventional risks, we finally constructed receiver operating characteristic (ROC) curves were finally constructed. The DeLong test was used to compare the area under the curve (AUC) between the models. R version 3.4.3 and Empower software were used for all statistical analyses. A two-sided P<0.05 was used to establish statistical significance.

## 3 Results

### 3.1 Initial characteristics of individuals

This study encompassed 11016 participants, characterized by an average age of 49.97 ± 17.45 years, with a slight majority of females (51.55%) over males (48.45%). The median TyG-BMI is 242.03, with a cardiovascular disease prevalence of 11.40%.

As detailed in **Table 1**, participants were categorized into tertiles based on TyG-BMI values. A significant trend emerged, with the weighted prevalence of CVD increasing across TyG-BMI tertiles (P<0.001). Furthermore, statistically significant variations were observed among the three tertiles in terms of gender, age, race, education level, PIR, alcohol consumption level, eGFR, hyperlipidemia, hypertension, and diabetes mellitus (P<0.001).

**Table 1:** Initial characteristics of individuals according to TyG-BMI tertiles.

N	Tertile 1	Tertile 2	Tertile 3	*p*-value
	3672	3672	3672	
Age(years)	45.07 (43.86,46.28)	50.41 (49.63,51.20)	49.45 (48.54,50.37)	<0.001
Male (%)	43.66 (41.11,46.25)	55.66 (54.06,57.26)	46.86 (44.57,49.18)	<0.001
Race (%)				<0.001
Mexican American	5.13 (4.08,6.43)	9.65 (7.91,11.71)	10.90 (8.76,13.48)	
Other Hispanic	5.68 (4.52,7.10)	6.99 (5.71,8.54)	6.91 (5.78,8.24)	
Non-Hispanic White	66.58 (63.24,69.76)	65.73 (62.47,68.86)	64.66 (60.71,68.42)	
Non-Hispanic Black	9.80 (8.33,11.49)	9.50 (7.89,11.39)	11.65 (9.57,14.12)	
Other Race	12.82 (11.10,14.76)	8.12 (6.82,9.66)	5.87 (4.82,7.13)	
Education (%)				<0.001
Less than high school	20.36 (18.28,22.60)	23.39 (21.01,25.95)	24.42 (22.41,26.55)	
High school or equivalent	67.11 (63.72,70.33)	61.72 (58.28,65.05)	59.72 (57.32,62.07)	
College or above	12.53 (10.58,14.79)	14.89 (13.12,16.84)	15.86 (14.34,17.52)	
PIR				<0.001
<1.3	18.56 (16.43,20.89)	18.47 (16.60,20.50)	22.33 (20.22,24.58)	
1.3-3.4	38.19 (35.70,40.74)	40.45 (38.02,42.92)	43.21 (40.55,45.92)	
≥3.5	43.26 (39.62,46.97)	41.08 (37.95,44.29)	34.46 (31.29,37.77)	
Smoker (%)	41.88 (39.12,44.69)	45.58 (42.88,48.31)	45.25 (43.02,47.50)	0.056
Drinking				0.015
Light drinking	46.78 (44.02,49.57)	50.43 (48.22,52.64)	44.67 (42.04,47.34)	
Moderate drinking	35.04 (32.88,37.25)	32.22 (30.23,34.28)	36.09 (33.99,38.25)	
Heavy drinking	18.18 (16.33,20.18)	17.35 (15.59,19.27)	19.24 (16.96,21.73)	
eGFR (mL/min/1.73 m2)	98.04 (96.65,99.42)	92.57 (91.52,93.61)	94.12 (93.00,95.24)	<0.001
hyperlipidemia (%)	41.19 (38.68,43.74)	65.31 (62.93,67.62)	78.37 (76.38,80.23)	<0.001
Hypertension (%)	25.81 (23.55,28.21)	43.20 (40.56,45.89)	56.52 (54.04,58.97)	<0.001
Diabetes (%)	4.58 (3.85,5.45)	13.46 (12.18,14.85)	29.38 (27.27,31.57)	<0.001
Cardiovascular disease (%)	6.65 (5.52,7.99)	9.69 (8.34,11.23)	12.33 (10.97,13.84)	<0.001

Means (95% CI) were used to represent continuous variables, while proportions (95% CI) were used to represent categorical data.

Because of the high percentage of missing values, **Supplementary Table S1** compares baseline demographic and health indicators for the excluded and included groups. There was no significant difference in the prevalence of cardiovascular disease between these two groups(P>0.05).

### 3.2 Association of the triglyceride glucose-body mass index with cardiovascular disease

**Table 2** demonstrates a significant positive association between TyG-BMI and CVD. This association remains robust when TyG-BMI is analyzed as a continuous or category variable, showing statistically significant association in unadjusted, partially adjusted, and fully adjusted models (P<0.001). Specifically, in the models, the third tertile of TyG-BMI consistently displayed a higher CVD prevalence than the first tertile. Notably, in the fully adjusted Model 3, the prevalence of CVD in the third tertile was 38% higher than in the first tertile [1.380(1.080,1.763), P=0.013]. The test for trend is also statistically significant in all models(P<0.05).

**Table 2:** The association between weighted TyG-BMI and cardiovascular disease.

Exposure	Model 1 OR (95CI%) P-value	Model 2 OR (95CI%) P-value	Model 3 OR (95CI%) P-value
TyG-BMI Continuous	1.004(1.003,1.005) <0.001	1.005(1.004 1.006) <0.001	1.003(1.002,1.005) <0.001
TyG-BMI Tertiles			
Tertile 1	1	1	1
Tertile 2	1.507(1.197,1.896) <0.001	1.195(0.937, 1.525) 0.156	1.045(0.794, 1.375) 0.756
Tertile 3	1.975(1.583,2.464) <0.001	1.922(1.563, 2.364) <0.001	1.380(1.080,1.763) 0.013
*P* for trend	<0.001	<0.001	<0.001

Model 1 was unadjusted for variables, model 2 was adjusted for sex, age, and race, and model 3 was adjusted for the above factors smoking, alcohol consumption, education, PIR, hyperlipidemia, diabetes, and hypertension.

**Figure 2:**
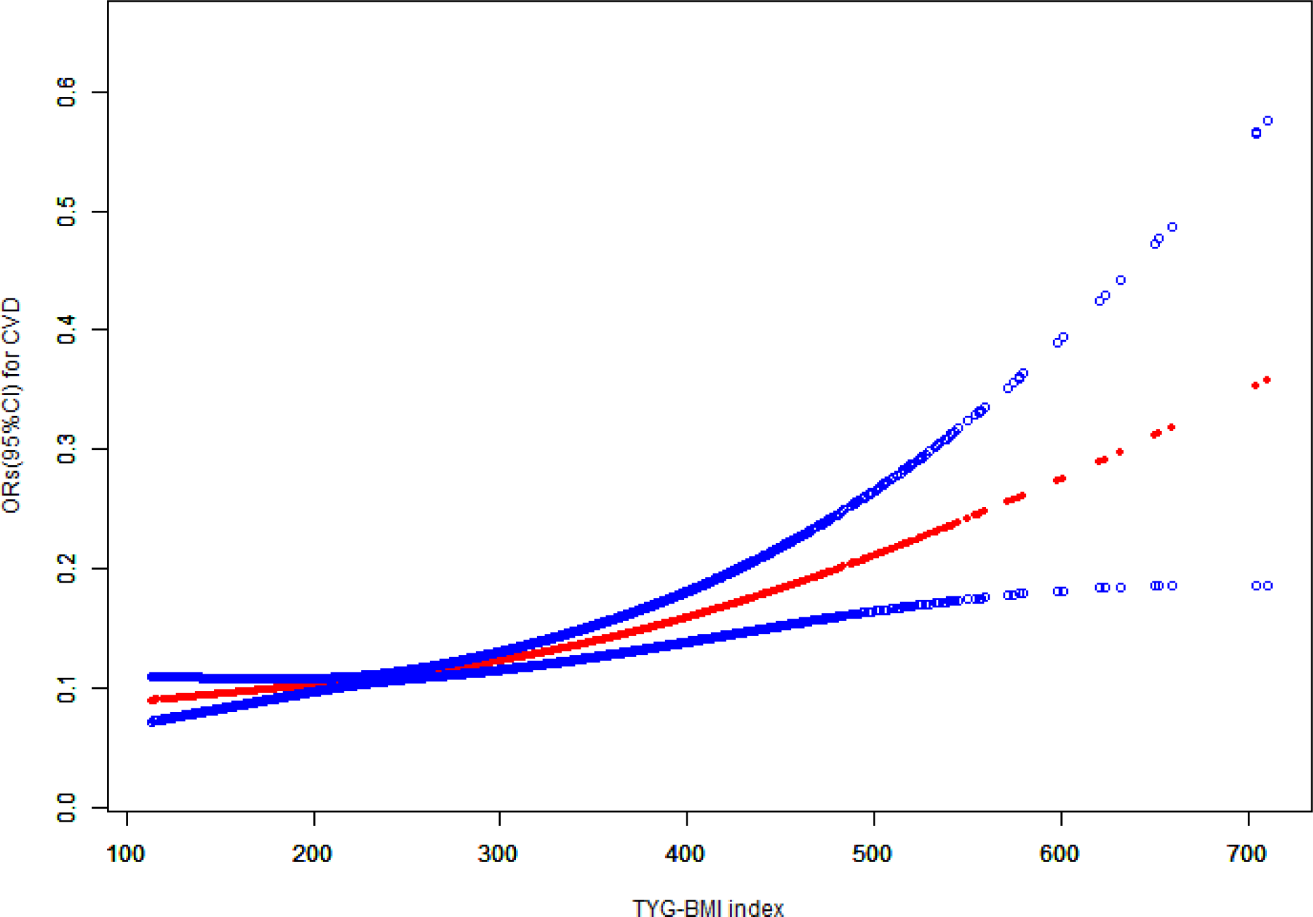
The association between TyG-BMI and cardiovascular disease. TyG-BMI, triglyceride glucose-body mass index; CVD, cardiovascular disease; CI, confidence interval; OR, odds ratio.

Additionally, **Figure 2** illustrates the nonlinear association between TyG-BMI and the prevalence of cardiovascular disease.

### 3.3 Subgroup investigation of the association between the triglyceride glucose-body mass index and cardiovascular disease

Subgroup analyses, based on factors such as age, gender, race, education, PIR, smoking, hyperlipidemia, hypertension, and diabetes were conducted to assess the potential association between the TyG-BMI and CVD. Interaction analysis showed that the positive association between TyG-BMI and cardiovascular disease was not affected by gender, race, education, PIR, smoking, hypertension, and diabetes factors (P>0.05). However, statistically significant differences were found between age, and hyperlipidemia subgroups (P<0.05) (**Table 3**).

**Table 3:** Subgroup analysis of the association between weighted TyG-BMI and cardiovascular disease.

Subgroup	OR (95%CI)	*P* Value	*P* for interaction
Gender			0.073
Male	1.005 (1.003, 1.007)	<0.001	
Female	1.002 (1.000, 1.004)	0.021	
Age			0.004
<50	1.005 (1.003, 1.008)	<0.001	
≥50	1.001 (1.000, 1.002)	0.069	
Race			0.383
Mexican American	1.001 (0.997, 1.005)	0.597	
Other Hispanic	1.001 (0.996, 1.005)	0.816	
Non-Hispanic White	1.003 (1.002, 1.005)	<0.001	
Non-Hispanic Black	1.004 (1.002, 1.006)	<0.001	
Other Race	1.005 (1.000, 1.009)	0.060	
Education level			0.070
Less than high school	1.001 (0.999, 1.003)	0.296	
High school or equivalent	1.005 (1.003, 1.007)	<0.001	
College or above	1.003 (1.001, 1.005)	<0.001	
PIR			0.968
<1.3	1.000 (0.999, 1.001)	0.841	
≥1.3 and <3.5	1.000 (0.999, 1.001)	0.860	
≥3.5	1.000 (0.999, 1.001)	0.898	
Smoking			0.771
Yes	1.004 (1.002, 1.005)	<0.001	
No	1.003 (1.001, 1.005)	0.002	
Hyperlipidemia			0.003
Yes	1.005 (1.003, 1.006)	<0.001	
No	1.000 (0.997, 1.003)	0.909	
Hypertension			0.386
Yes	1.004 (1.002, 1.005)	<0.001	
No	1.002 (1.000, 1.005)	0.048	
Diabetes			0.057
Yes	1.005 (1.003, 1.007)	<0.001	
No	1.002 (1.000, 1.004)	0.037	

### 3.4 Sensitivity analysis

In **Table 4**, we performed sensitivity analyses using unweighted logistic analyses, TyG-BMI was positively associated with the prevalence of cardiovascular disease in all models, both as a continuous and categorical variable(P<0.05). These findings are consistent with those reported in **Table 2**.

**Table 4:** The association between TyG-BMI and cardiovascular disease in a sensitivity analysis using unweighted logistic regression analysis.

	Model 1	Model 2	Model 3
	OR (95CI%) *P*-value	OR (95CI%) *P*-value	OR (95CI%) *P*-value
TyG-BMI Continuous	1.004 (1.003, 1.004) <0.001	1.005 (1.004, 1.005) <0.001	1.003 (1.002, 1.004) <0.001
TyG-BMI Tertiles			
Tertile 1	1	1	1
Tertile 2	1.514 (1.297, 1.768) <0.001	1.218 (1.031, 1.439) 0.020	1.085 (0.912, 1.291) 0.357
Tertile 3	1.854 (1.596, 2.155) <0.001	1.825 (1.549, 2.151) <0.001	1.311 (1.092, 1.573) 0.004
P for trend	<0.001	<0.001	0.003

Model 1 was unadjusted for variables, model 2 was adjusted for sex, age, and race, and model 3 was adjusted for the above factors smoking, alcohol consumption, education, PIR, hyperlipidemia, diabetes, and hypertension.

### 3.5 Incremental effect of the triglyceride glucose-body mass index for predicting cardiovascular disease

**Figure 3:**
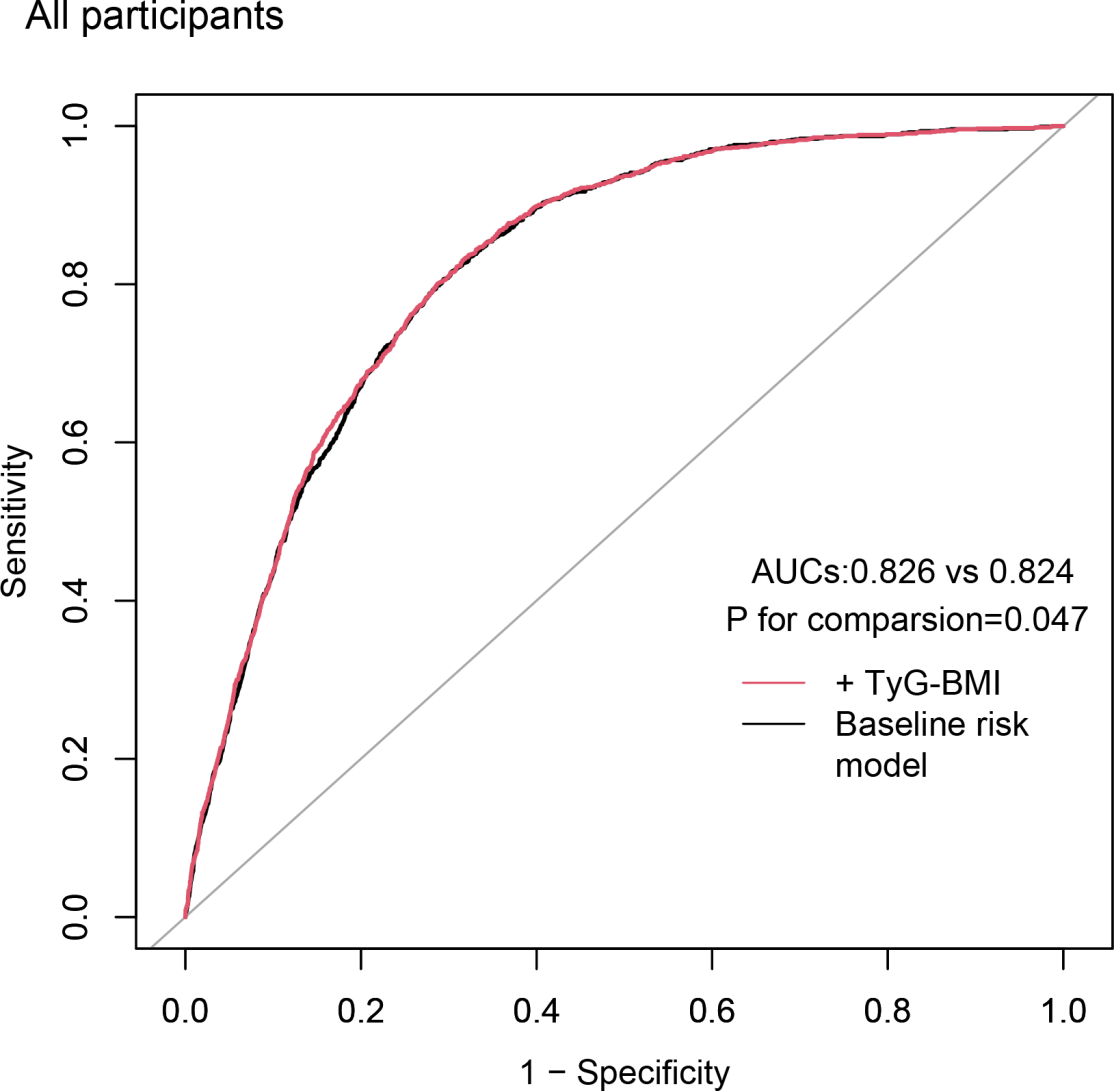
Incremental effect of the TyG-BMI for predicting CVD. The baseline risk model includes age, gender, smoking, hyperlipidemia, diabetes, and hypertension; TyG-BMI, triglyceride glucose-body mass index; CVD, cardiovascular disease; AUC, area under curve.

We evaluated baseline risk model ROC curves for all participants, including traditional CVD risk factors (age, gender, smoking, hyperlipidemia, diabetes, and hypertension) as well as the TyG-BMI index. The baseline risk model showed an AUC of 0.824, while the model including the TyG-BMI index showed an AUC of 0.826. This difference was statistically significant (p = 0.047) (**Figure 3**).

## 4 Discussion

To the best of our knowledge, this study is the first to explore the relationship between the prevalence of cardiovascular disease and the novel biomarker TyG-BMI. Using data from 11,016 participants in the NHANES database from 2011 to 2020, we found that TyG-BMI levels were significantly associated with an increased prevalence of cardiovascular disease, both as a continuous and categorical variable (P<0.05). This association persisted even after adjusting for basic demographic characteristics, lifestyle factors, and prior medical history. Furthermore, the robustness of our findings was confirmed through sensitivity analyses, which yielded results consistent with the weighted analyses (P<0.05).The TyG-BMI value has been validated by earlier studies as a trustworthy proxy indicator for IR (22–24). Given the public health implications of IR and CVD (25), recent investigations have linked IR to inflammation, oxidative stress, altered microRNA expression, disruptions in insulin metabolism signaling pathways, and mitochondrial dysfunction. These factors contribute to various cardiovascular risks, including lipid disorders, coronary artery disease, hypertension, vascular calcification, heart failure, and cardiomyopathy (26–29). The study by Yamazoe independently links the prevalence of coronary artery calcification and IR (29). In contrast, a sizable prospective cohort research which followed up for 5.5 years, shows that in 17.9% of participants with diabetes, it is linked to an increase peripheral in artery disease and heart failure (30). Moreover, a well-established link exists between IR and atherosclerosis (31), a key CVD risk factor, with the TyG-BMI index as an effective CVD predictor. A study by Yi Hu indicates an L-shaped association between TyG-BMI and all-cause mortality in patients with atrial fibrillation admitted to the ICU (15). According to surveys, individuals with heart failure can also utilize the TyG-BMI index to predict their one-year all-cause mortality (32). Additionally, a higher TyG-BMI index has been linked to prehypertension in Chinese adults (33). In our research, according to the fully adjusted model of our analysis, compared to individuals in the lowest tertile, those in the greatest TyG-BMI tertile exhibited a substantially greater likelihood of cardiovascular disease (OR=1.380; 95%CI=1.080,1.763). The adverse effects of IR on cardiovascular health are highlighted by this research. Moreover, interaction studies indicate that the positive association between the TyG-BMI value and CVD was not substantially affected by gender, race, education level, PIR, smoking, hypertension, hyperlipidemia, and diabetes (all P for interaction > 0.05). The ROC curves showed that the model with the addition of TyG-BMI had an incremental effect on predicting the prevalence of cardiovascular disease. However, A retrospective study indicated that a high TyG-BMI was positively associated with the incidence of major adverse cardiac and cerebrovascular events (MACCEs). it did not enhance the predictive power for MACCEs in elderly patients and female patients (34). The discrepancy between the results of the two studies may arise from differences in the populations and outcome variables. Our study focused on the general U.S. adult participants, and CVD defined using data from the NHANES database questionnaire. In contrast, the retrospective study examined patients in Henan Province, China, who underwent Percutaneous coronary intervention (PCI) and were treated with drug-eluting stent (DES), with the outcome defined as a composite of MACCEs at follow-up.

However, in our subgroup analysis of this study, age, and hyperlipidemia significantly influence the association between TyG-BMI and the prevalence of cardiovascular disease (*P* for interaction <0.05). In younger individuals, the TyG-BMI may better reflect cardiovascular risk due to their heightened sensitivity to metabolic changes (35). Conversely, in older adults, the presence of more significant cardiovascular risk factors may attenuate the impact of TyG-BMI. Hyperlipidemia, an independent risk factor for CVD, is closely associated with IR and metabolic syndrome (36), thereby enhancing the predictive power of TyG-BMI. Therefore, additional studies are required to validate these associations and investigate the underlying mechanisms. The advantages of our study include the use of the NHANES database, known for its large sample size and representativeness, and adjustments for some confounding covariates, enhancing the reliability of our results. We also performed subgroup analyses and sensitivity analyses to further investigate the robustness of the association between TyG-BMI index and cardiovascular disease (CVD). Nevertheless, there are limitations to acknowledge. Firstly, Because of our cross-sectional methodology, it is not feasible to establish a causal association between the TyG-BMI values and the prevalence of cardiovascular disease. Secondly, participants with missing TyG-BMI data were excluded to maintain data completeness. The **Supplementary Table** presents a comparison of baseline characteristics between the included and excluded groups. Additionally, weighted and sensitivity analyses were conducted to mitigate potential bias. Despite these efforts, the exclusion of these participants may still impact the generalizability of our findings. Thirdly, residual confounding cannot be ruled out despite adjusting for multiple covariates, including potential medication effects or other comorbidities. Finally, certain variables potentially relevant to CVD were unavailable in the NHANES dataset, possibly impacting result accuracy. Therefore, to support and build on our findings, further extensive prospective studies involving several centers should be conducted.

## 5 Conclusion

The TyG-BMI provides a practical approach to assessing cardiovascular disease risk in adults. Its simplicity and accessibility highlight its potential clinical value. However, prospective studies are necessary to validate its predictive reliability and explore broader applications.

## Data availability statement

The datasets presented in this study can be found in online repositories. The names of the repository/repositories and accession number(s) can be found in the article/**Supplementary Material**.

## Ethics statement

The studies involving humans were approved by NCHS Ethics Review Board, the ERB is affiliated with the National Center for Health Statistics. The studies were conducted in accordance with the local legislation and institutional requirements. The participants provided their written informed consent to participate in this study. Written informed consent was obtained from the individual(s) for the publication of any potentially identifiable images or data included in this article.

## Author contributions

RW: Software, Methodology, Investigation, Data curation, Conceptualization, Writing – review & editing, Writing – original draft. XC: Writing – review & editing, Supervision, Methodology, Funding acquisition. WT: Writing – review & editing, Supervision, Methodology.
