# Supplementary material for: Association between triglyceride glucose body mass index and cardiovascular disease in adults: evidence from NHANES 2011- 2020
Source: Front Endocrinol (Lausanne). 2024 Jul 16;15:1362667. doi: 10.3389/fendo.2024.1362667 (PMC11286411; doi:10.3389/fendo.2024.1362667)
Supplement: Supplementary file 1 [file Table_1.docx]

**Table S1 Baseline Characteristics of the Excluded and Included Groups**

|  | Excluded Group | Included Group | *p*-value |
| --- | --- | --- | --- |
| N | 15247 | 11033 |  |
| Age(years) | 47.61 (46.97,48.25) | 48.29 (47.62,48.96) | 0.035 |
| Male (%) | 47.79 (46.89,48.69) | 48.69 (47.46,49.93) | 0.235 |
| Race (%) |  |  | 0.012 |
| Mexican American | 8.43 (6.93,10.22) | 8.53 (7.02,10.34) |  |
| Other Hispanic | 6.90 (5.75,8.27) | 6.52 (5.46,7.79) |  |
| Non-Hispanic White | 63.38 (59.71,66.90) | 65.63 (62.49,68.65) |  |
| Non-Hispanic Black | 12.17 (10.32,14.29) | 10.34 (8.76,12.18) |  |
| Other Race | 9.12 (7.94,10.45) | 8.97 (7.89,10.18) |  |
| Education (%) |  |  | 0.449 |
| Less than high school | 13.71 (12.39,15.14) | 14.41 (12.98,15.98) |  |
| High school or equivalent | 23.56 (22.01,25.18) | 22.70 (21.17,24.30) |  |
| College or above | 62.74 (60.49,64.93) | 62.89 (60.50,65.21) |  |
| PIR |  |  | 0.618 |
| <1.3 | 20.15 (18.50,21.91) | 19.79 (18.17,21.51) |  |
| 1.3-3.4 | 41.29 (39.65,42.94) | 40.60 (38.88,42.35) |  |
| ≥3.5 | 38.56 (36.26,40.91) | 39.61 (37.03,42.25) |  |
| Smoker (%) | 42.59 (41.16,44.04) | 44.20 (42.52,45.91) | 0.059 |
| Drinking |  |  | 0.445 |
| Light drinking | 47.13 (45.80,48.46) | 47.30 (45.60,49.00) |  |
| Moderate drinking | 35.48 (34.37,36.60) | 34.45 (33.07,35.85) |  |
| Heavy drinking | 17.39 (16.22,18.63) | 18.25 (17.00,19.58) |  |
| eGFR (mL/min/1.73 m2) | 94.03 (93.21 ,94.85) | 94.93 (94.10 ,95.76) | 0.015 |
| hyperlipidemia (%) | 51.83 (50.27,53.39) | 61.44 (59.98,62.88) | <0.001 |
| Hypertension (%) | 32.12 (30.82,33.44) | 33.65 (32.00,35.35) | 0.100 |
| Diabetes (%) | 13.03 (12.23,13.87) | 15.72 (14.61,16.90) | <0.001 |
| Cardiovascular disease (%) | 8.97 (8.35,9.63) | 9.54 (8.66,10.49) | 0.2514 |

Means (95% CI) were used to represent continuous variables, while proportions (95% CI) were used to represent categorical data.
